# Natural Language Processing Based Instrument for Classification of Free Text Medical Records

**DOI:** 10.1155/2016/8313454

**Published:** 2016-09-07

**Authors:** Manana Khachidze, Magda Tsintsadze, Maia Archuadze

**Affiliations:** Ivane Javakhishvili Tbilisi State University, University St. 3, 0179 Tbilisi, Georgia

## Abstract

According to the Ministry of Labor, Health and Social Affairs of Georgia a new health management system has to be introduced in the nearest future. In this context arises the problem of structuring and classifying documents containing all the history of medical services provided. The present work introduces the instrument for classification of medical records based on the Georgian language. It is the first attempt of such classification of the Georgian language based medical records. On the whole 24.855 examination records have been studied. The documents were classified into three main groups (ultrasonography, endoscopy, and X-ray) and 13 subgroups using two well-known methods: Support Vector Machine (SVM) and *K*-Nearest Neighbor (KNN). The results obtained demonstrated that both machine learning methods performed successfully, with a little supremacy of SVM. In the process of classification a “shrink” method, based on features selection, was introduced and applied. At the first stage of classification the results of the “shrink” case were better; however, on the second stage of classification into subclasses 23% of all documents could not be linked to only one definite individual subclass (liver or binary system) due to common features characterizing these subclasses. The overall results of the study were successful.

## 1. Introduction

Almost 50 years have passed since the first medical record processing systems have been developed [1]. These systems formed the basis for several directions in modern medical informatics and played a significant role in the EMR system development, as well as in its integration into the health systems of different countries [2].

Classification is done using various algorithms while their effectiveness is evaluated using such values as precision, recall, accuracy, and *F* measure [3].

It has to be mentioned that the structure of the database (the volume, data types, feature selection methods, etc.) plays a significant role in the evaluation of the above-mentioned characteristics. There are a number of methods of classification: classification performed manually, classification with the help of a system based on rules and vocabularies, automated classification using IR methods, machine learning algorithms, and the so-called hybrid systems combining several classification methods [4].

In 2011 the Ministry of Labor, Health and Social Affairs of Georgia introduced a Health Management Information System in order to interconnect the information needs of the Ministry, insurers, providers, and patients. One of the main modules of this system is the “electronic medical record.”

It is stated in Georgia's Health Management Information System Strategy that “new software products such as electronic medical record, personal health record, and public health surveillance system will be prepared and integrated.”

Seeing that these systems are to comply with the requirements and standards set by the government, they will allow for a uniform set of core health data elements to be collected throughout the healthcare delivery system and should be focused on clinical data. The main target of the Health Management Information System is to collect clinical information, improve care/information delivery to individual patients, and aggregate the population's healthcare information into a single whole. Such a fully functional system will contain all the information on medical aid provided to a patient earlier, currently and in future. So, instead of fragmentary information existing in present we shall obtain a full time-line report of each patient's medical history. In order to facilitate processing and rationalization of the patient's clinical and demographic data, the system will perform and present identification of his/her medical problem(s) and give a list of prescribed medications together with the patient's vital data, medical characteristics, medical problem(s), immunization, and lab and X-ray diagnostic results.

The existing large-scale health management organizations and providers in Georgia have internal information systems of their own used for patient-doctor relations management and for registering only essential personal data. Providers store all the information on the patient's medical history, such as lab/X-ray tests, electronically in the form of free text files or, in very rare cases, in the form of structural data of the internal information system. As has been mentioned above the implementation of the Health Management Information System is planned for the nearest future, and after the introduction of the “electronic medical record” information system, all healthcare organizations are incumbent to comply with all the standards provided by the government and to send (according to notifications and the data exchange standards) all clinical data in the form of unified standards approved by the Ministry of Labor, Health and Social Affairs of Georgia.

The problem of structuring and classification of documents (free text files) containing information on services provided earlier, including medical history, needs to be tackled. The presented study deals with the process of software development used to classify/structure some types of medical records in order to fit them into the main electronic medical records system provided by the Ministry Labor, Health and Social Affairs. Our work introduces the instrument for Georgian language based medical records classification that might be used as a separate module of data (already existing electronic medical records) import at Health Management Information System.

## 2. Materials and Methods

The material under processing is the so-called “medical records domain” which contains documents describing instrumental diagnostics records of one of Georgia's health providers over the period from 2012 until 2014. The documents are presented in a small volume (no more than 300–350 words) Free Text (free text  .doc or  .docx format) form. They contain various ultrasonography, X-ray, and endoscopy diagnostic records. The numbers of records refer to one and the same patient: some of them indicate different types of research or same studies repeated at another time. The ultrasonography domain covers various organs (liver and the biliary system ultrasonography, kidney and the urinary system ultrasonography, gynecologic ultrasound, thyroid, breast ultrasound, and vascular Doppler ultrasound), the total of 12.864 examination records. X-ray pictures contain images of the body (chest, abdomen, spine, limbs, esophagus and stomach, and X-ray studies of large and small bowels), totally 10.523 study entries. As for endoscopy, 1.468 records were presented (Table 1). There are 500–1.000 various subgroup records excluding endoscopy, where no subgroup is presented.

### 2.1. Methods

Classification of the medical records may be considered as a particular case of information retrieval (IR), text classification, and the machine learning algorithms for nonstructured electronic records database can be applied. Natural Language Processing (NLP) is best adopted and applicable for solving such a problem [5–7]. According to the results of various research groups working in bioinformatics, it is one of the most common approaches to nonstructured medical data processing [8–10].

Classification covers the process of feature selection, especially if diagnosis is done in the natural language according to the above-described symptoms. Combination of machine learning algorithms with feature selection techniques is quite common [11].

It is not surprising that manual classification takes longer than its automated analogs; for instance, manual classification of 5.232 medical records took 176 hours while the same process performed automatically was completed in 4.5 hours [12].

Research shows that the effectiveness of machine learning algorithms also varies according to the disease classification task.

Combination of information retrieval and machine learning was successfully used for medical database classification at the Brazilian Pediatric Healthcare Institution [13].

Out of all known algorithms the SVM is one of the most common and popular especially for small size text processing [14]. SVM used for classifications of such diseases as cancer and diabetes produces quite good results [15–17].

The SVM and KNN combination was successfully used for Multiclass Text Classification 2008, and some good results were achieved in 2010 by joint scheme of GA and KNN. According to the results of leading scientists in IR, statistical methods are quite promising even without any morphological analysis of the language 2001, 2011.

Compared with other languages, research performed in Georgian language based text classification issues is in the range from none to very little. Georgian is an agglutinative language (with up to eight morphemes per word), but it is an exception: it has a high number of irregular verbs requiring different formations. There are also certain prefixes and suffixes joined together to build a verb. In some cases there can be up to eight different morphemes in one verb at the same time. One can judge the complexity of the Georgian verbal system according to the term “screeve” (the set of six verb forms inflected for person and number) used by linguists. The Georgian word “chageshenebinat” meaning “you (plural) had built in” breaks down to parts: ch-a-g-e-shen-eb-in-a-t. Each morpheme here contributes to the meaning of the verb tense or the person who has performed the action. The verb conjugation also exhibits polypersonalism: a verb may potentially include morphemes representing both the subject and the object. Verbs are mainly divided into four types: transitive verbs, intransitive verbs, verbs with no transitive counterparts, and indirect verbs. Each type uses different strategies to build the verb complex. The form-processing of noun in Georgian is easier; it has only one root, but declension is applied. The noun declension depends on the ending of the root: if it ends with a vowel, the declension can be either truncating (roots ending with -e or -a) or nontruncating (roots ending with -o or -u). In truncating declensions, the last vowel of the word stem is lost in the genitive and the instrumental cases [18].

The Georgian nominal has a series of morpheme slots that must be filled in a specific order: noun root + plural suffix + case suffix (+ postposition).

The task of document classification is based on the text indexation performed using stemming—commonly used in information retrieval but it is also beneficial in machine learning based models applications as it improves the system quality. Keeping in mind the structure of the language we decided to use a new stemming algorithm developed specifically for the Georgian language [19, 20] in text-processing initial module of our system and then apply KNN and SVM.

Our task can be considered as a junction of four different problems:

- Text preprocessing (deidentification).
- Initial processing of text—tokenization and lemmatization—according to characteristics of the Georgian language.
- Weighting scheme and feature selection.
- Classifiers (classification of texts): medical records classification/structuring so that they can be submitted to electronic medical records system according to the type of the research performed.

Let us describe each task separately.

#### 2.1.1. Text Identification according to Patient

As documents used for classification contain data of personal character (information on patient and doctor) deidentification is required. Analysis of document forms has revealed that in the vast majority of records only first name and last name of patient are used for his/her identification. In the document referred to there was no indication of the patient's personal ID or the insurance ID. All documents contain the record on the patient's age and/or date of birth along with the date/time of the tests performed.

**Figure 1:**
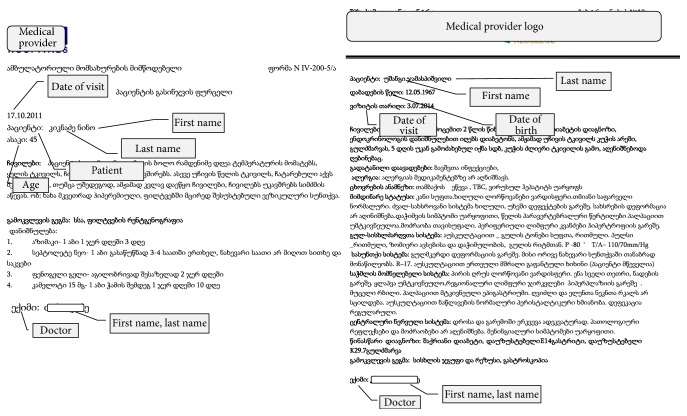
Example of medical record form.

The identification fields in the records under review were presented in the form shown in Figure 1.

Patient identification information is provided at the very beginning of the text while the name of doctor appears at very end of the document. For deidentification of texts we are deleting the last line and the first several lines according to the following rule: the words “age,” “date of birth,” and “date/time” are tracked along with their line numbers where they appear first. The greatest line number (but less or equal to 5, as the maximum number of lines used for person identification is 5) defines the amount of lines to be discarded from the text.

#### 2.1.2. The Initial Processing of the Text (Tokenization and Lemmatization)

There may be different ways of text processing [21]. For processing medical texts the Unified Medical Language System (UMLS) [22, 23] is used, especially in machine learning processes. The use of this system in our case is complicated because of lack of English equivalents of Georgian words. Almost all well-known categorization algorithms use text minimization approach leaving in it only the most frequently recurring words. Word processing is necessary to calculate the frequency of words recurring in different forms in order not to count them as different. Word processing means leaving the part which remains unchanged in speech [24]. The most famous algorithm nowadays—the Porter algorithm—solves the problem successfully, but it is useless in case of the Georgian language as its grammar structure is completely different from that of the English language [25, 26].

A new method [27] developed in the framework of the Tbilisi State University (TSU) target research project was applied at the initial stage of text processing as each text used either in the process of classification or in the process of system testing is subject to the full development process which includes the following:

1. Creating a list of the so-called “stop-words” (for the Georgian language these are conjunctions, interjections, pronouns, etc.) and their replacement with the “wildcard” characters, where the numeric symbols are withdrawn as well.
2. Identification of words represented in non-Georgian characters and their replacement with Georgian analog words (some terms in medical records contain medical terms written in English).
3. The suffix stripping or stemming operation: to remove the longest one from all the available suffixes list for Georgian language based texts.

It allows to group (enabling word counting in the text) the same lexical units in the text despite the diversity of their forms in process of speech. Unlike the Porter algorithm, this method uses the words database (containing almost 60 suffixes as well) to determine the root. However, the application of this word database for medical texts root determination led to a number of problems: recall of database in frames of medical terms, reliability of database (according to the meaning of terms), and database update. In order to overcome these difficulties we updated the Georgian words database [28] with more nouns and terms used in Georgian medical terminology according to ICD-10- (the 10th revision of the International Statistical Classification of Diseases and Related Health Problems (ICD), a medical classification list by the World Health Organization (WHO)).

#### 2.1.3. Weighting Schema and Feature Selection

The result of initial processing of a text is an indexed document that is presented as a vector in the feature space. This vector contains such elements as weights in the appropriate couples “term/weight.” Weights are calculated in framework of the tf-idf scheme (term frequency-inverse document frequency) [29]:

(1) $$\begin{array}{c} w_{i,j}=tf_{i,j}\times \log\frac{N}{{df}_{i}}, \end{array}$$

where *w*_*i*,*j*_ is *i*th term weight in *j*th document; tf_*i*,*j*_ is *i*th term frequency in *j*th document; *N* is the number of documents in the collection; df_*i*_ is the document frequency of *i*th term in the collection.

It is clear that along with the raised document number the word vocabulary increases as well [30]. Thus, the feature space increases and in order to decrease it the term elimination is used.

In order to shrink the term space, various methods are applied. In the majority of cases the most frequently used terms or the most important ones are left/preserved while others are ignored/discarded. Thus, the calculation of term frequency is crucial [31]. In order to reduce dimensions of the feature space we used the approach that considers special characteristics of the Georgian language. The number of stop-words in the text provided was very small (≪1%). While processing the three class fourteen subclass documents we discovered that, in the space of each subclass defining term space, there were several similar terms for all three classes (the total number of these terms is 21). The subclass vector analysis revealed that the number of the same terms for ultrasonography was equal to 10 (location, shape, contour, structure, organ, indicator, size, tissue, data, and hub), and for the X-ray the number was 17 (tissue, structure, shape, contour, body, indicator, fluid, spot, size, data, hub, muscle, bone, image, density, change, and structure). We call these words the “pseudo stop-words” and discarded them from the feature space. Let us call its appropriate vector the “shrink” vector.

#### 2.1.4. Classifiers

While choosing the most appropriate machine learning algorithm to tackle classification problem we faced, we considered both generative and discriminative models [32]. Classifier selection primarily depends on the data to be classified. Discriminative models are usually preferred, but for our task it is crucial to define the connection between features. And as generative models fail to fulfill this task (for instance, Naive Bayes disadvantage is that it cannot learn interactions between features), we decided to go with discriminative model and chose KNN (for simplicity) and SVM (for power and accuracy) for the processing. Let us shortly discuss the classifiers chosen.

*KNN*. The KNN rule is a very simple method that classifies unlabeled data based on their similarity to the examples in the training dataset. To “guess” the label of a new instance the KNN algorithm will find the *K* closest neighbors to the new instance from the training data, and the guessed class label will then be set as the most common label among the *K* closest neighbor [24].

The KNN only requires *K* (an integer), set of labeled training data, and a system or standard of measurement to define the “closeness”; however, it can be slow if there is a large number of training examples, but it is useful for short texts as it memorizes all examples in the training dataset and then compares them with the test document.

*SVM (SVMs)*. SVM analyzes data used for classification and assigns a new example to only one category (class). In SVM presentation documents are considered to be points in a space, mapped in such a way that different class documents are divided by a certain gap (wide as possible). When a new example document is provided, it is mapped into that same space and based on which side of the gap it falls, it is assigned to an appropriate category. The main idea of SVM is to find out the linear separating hyperplane—the optimal separating hyperplane which maximizes the margin (gap) [33].

SVM is a powerful classifier that works well on the wide range of classification problems, it captures the inherent characteristics of the data better, and it is able to learn independent of the dimensionality of the feature space and with high accuracy. SVM is especially popular for solving text classification problems where high-dimensional spaces are common. But it is also memory-intensive and choosing the kernel might be a headache.

Using the above-mentioned classifiers a text was classified in two stages: during the first stage, categorization was done according to the research performed (ultrasonography, X-ray, and endoscopy; note: each document may be assigned to one class only). After this stage the categorization proceeds according to the defined class subclasses (in case of ultrasonography 7 subclasses are defined, for the X-ray we have 6, and no subclasses are assigned for endoscopy).

As mentioned above in order to represent a document as a space vector in the feature space, we introduced some changes; more precisely, we got fewer terms by discarding the “pseudo stop-words.”

Thus, the machine learning process was performed using both the classic document vector (with all features) and the “shrink” (with discarded features) one. The results obtained are presented in Tables 3 and 4.

## 3. Results and Discussion

In the present study we considered the problem of classification of medical texts using two well-known methods: KNN and SVM. The initial processing of texts was carried out identically for both methods (Table 2). The results thus obtained are presented in: Table 2 (*R*: Recall, *P*: precision, *F*: *F* measure, ERR: error rate, and Acc: accuracy).

SVM shows better results; thus, the feature selection methods (two forms: classic case and shrink case with and without “pseudo stop-words”) have been tested only for SVM.

In the process of machine learning during the first stage of classification, the documents appropriate for each class were presented with vectors comprising 21 elements less and for second stage classification with vectors comprising less than 10 and 17 elements, respectively.

The results have revealed that during the first stage of classification both methods of feature selection (classic and “shrink”) work successfully with quite close fitting results (Tables 3 and 4). It was clear that every document tested was assigned to only one class of instrumental examination except for liver and biliary system examination and contained data where 23% of all the documents failed to be linked to only one definite individual subclass due to common features of these above-mentioned subclasses. At the first stage of classification the results of machine learning obtained in the space of “pseudo stop-words” (shrink case) were better (Table 3) than the same studies in the space of ordinary characteristics (classic case) (Table 4).

The division of documents into subclasses at the second stage of classification was mainly unambiguous, excluding the results of research conducted for liver and biliary system examination (Table 5).

It should be noted that the results obtained at the second stage of classification for classic case (Table 5) were better than for the shrink case. Notwithstanding, we still believe that the shrink space of characteristics may be useful for a number of classification tasks where the nature of classes varies. Finally we may conclude that the SVM method used for classification of Georgian texts based on medical documents proved to be a little better than the KNN, but on the whole, application of both methods may be regarded expedient.

While SVM shows better result, the feature selection methods (two forms: classic case and shrink case with and without “pseudo stop-words”) have been tested only for SVM, but for sure KNN application is possible as well.

As to whether the proposed scheme might be applied to other languages or not, we think other agglutinative languages can use it with appropriate database and suffixes list, but there are some easier schemes (as we mentioned earlier, the Georgian language is an exception from agglutinative languages and it makes it harder for processing).

In order to fill in the fields in the electronic medical record system with data of earlier provided instrumental research, it is not sufficient to define its appropriate class or subclass. It is important to define the chronology of instrumental tests and identity of the patient. The reidentification of personal information is needed as well. To comply with rules of personal data security and at the same time to identify the multiple health records of the same patient, a different approach for deidentification is required. We are considering adding a separate module to our system for this task which will first group data according to patient (the age or date of birth along with name and surname will be used for identification) under the unique ID number (generated) and implement deidentification after that, and at the stage when reidentification is required the generated ID number will be applied.

## 4. Conclusions

The task of text classification is not new and research in this direction is quite intense, although it is mainly conducted on the English language based data. There are a number of studies devoted to other languages, while data processing based on the Georgian language is quite new.

Our work introduces the instrument for medical records classification based on the Georgian language that might be used as a separate module of electronic medical records import at Health Management Information System provided by the Ministry Labor, Health and Social Affairs. Two classifiers SVM and KNN were applied. According to the results obtained, both machine learning methods performed successfully with a little supremacy of SVM. In the process of classification a “shrink” method, based on features selection, was introduced and applied (SVM classifier). At the first stage of classification (ultrasonography, X-ray, and endoscopy group's classification) the results for the “shrink” case were better: *F* measure equals 0.914, 0.933, and 0.878 for ultrasonography, X-ray, and endoscopy, respectively, while the same studies performed on the space of ordinary characteristics (classic case) lead to inferior results of *F* measure (0.851, 0.852, and 0.877). However, during the second stage of classification into subclasses due to common characteristics of liver and biliary system examination data, 23% of all the documents failed to be linked to only one definite individual subclass; thus, results gained for classic case were better than for the “shrink” one (Table 5).

The used method might be considered for application not only for classification of Georgian language based medical data. Any kind of Georgian language based texts will do as well, but previous analysis of text will be recommended in order to define feature selection “shrink” method benefits. Future update of the database with appropriate theme words will also lead to better results.

## Figures and Tables

**Table 1:** The data.

Data	Total	Training data	Testing data
*Ultrasonography*	12.864	7.720	5.144
Liver		1.360	784
Biliary system		1.227	957
Kidney and urinary system		896	536
Gynecologic		1.372	942
Thyroid		1.101	851
Breast		771	531
Vascular Doppler		993	543

*X-ray*	10.524	5.262	5.262
Chest		849	849
Abdomen		1.057	1.057
Spine		946	946
Limbs		612	612
Esophagus and stomach		1.224	1.224
Large and small bowels		574	574

*Endoscopy*	1.468	734	734

*Total*			
Document number (ultrasonography, X-ray, endoscopy)	24.856	13.716	11.140

**Table 2:** Results of SVM versus KNN (feature selection classic method).

Feature selection classic method											
Class name	Subclass name	SVM					KNN				
		*R*	*P*	*F*	Acc	ERR	*R*	*P*	*F*	Acc	ERR
*Ultrasonography*		*0,91*	*0,80*	*0,85*	*0,85*	*0,15*	*0,83*	*0,81*	*0,82*	*0,84*	*0,17*
	Liver	0,75	0,53	0,62	0,86	0,14	0,72	0,51	0,60	0,85	0,15
	Biliary system	0,57	0,44	0,50	0,78	0,22	0,56	0,40	0,46	0,76	0,24
	Kidney and urinary system	0,94	0,81	0,87	0,97	0,03	0,78	0,59	0,67	0,92	0,08
	Gynecologic	0,91	0,87	0,89	0,96	0,04	0,70	0,63	0,66	0,87	0,13
	Thyroid	0,83	0,79	0,81	0,94	0,06	0,81	0,71	0,76	0,91	0,09
	Breast	0,90	0,77	0,83	0,96	0,04	0,87	0,59	0,70	0,92	0,08
	Vascular Doppler	0,87	0,72	0,79	0,95	0,05	0,87	0,70	0,77	0,95	0,05

*X-ray*		*0,89*	*0,81*	*0,85*	*0,85*	*0,15*	*0,81*	*0,79*	*0,80*	*0,81*	*0,19*
	Chest	0,89	0,85	0,87	0,96	0,04	0,78	0,84	0,81	0,94	0,06
	Abdomen	0,86	0,76	0,81	0,92	0,08	0,76	0,64	0,70	0,87	0,13
	Spine	0,89	0,77	0,83	0,93	0,07	0,78	0,63	0,70	0,88	0,12
	Limbs	0,99	0,77	0,87	0,97	0,04	0,83	0,57	0,68	0,91	0,09
	Esophagus and stomach	0,91	0,88	0,89	0,95	0,05	0,69	0,64	0,66	0,84	0,16
	Large and small bowels	0,91	0,94	0,93	0,98	0,02	0,84	0,66	0,74	0,94	0,07

*Endoscopy *		*0,97*	*0,80*	*0,88*	*0,98*	*0,02*	*0,93*	*0,75*	*0,83*	*0,97*	*0,03*

**Table 3:** Retrieval analysis (shrink case/SVM).

Calculation formulas							tp/(tp + fn)	tp/(tp + fp)	2*∗P∗R*/(*R* + *P*)	(tp + tn)/(tp + fp + fn + tn)	(fp + fn)/*n*
Medical tests performed	Total testing documents	Retrieved	True positive (tp)	False positive (fp)	False negative (fn)	True negative (tn)	*R*	*P*	*F*	Acc	ERR
Ultrasonography	11.140	5.802	5004	798	140	5.198	0,973	0,862	0,914	0,916	0,084
X-ray		5.478	5010	468	252	5.410	0,952	0,915	0,933	0,935	0,065
Endoscopy		872	705	167	29	9.701	0,960	0,808	0,878	0,982	0,018

**Table 4:** Retrieval analysis (classic case/SVM).

Calculation formulas							tp/(tp + fn)	tp/(tp + fp)	2*∗P∗R*/(*R* + *P*)	(tp + tn)/(tp + fp + fn + tn)	(fp + fn)/*n*
Medical tests performed	Total testing documents	Retrieved	True positive (tp)	False positive (fp)	False negative (fn)	True negative (tn)	*R*	*P*	*F*	Acc	ERR
Ultrasonography	11.140	5.798	4.657	1.141	487	4.855	0,905	0,803	0,851	0,854	0,146
X-ray		5.784	4.703	1.081	559	4.797	0,894	0,813	0,852	0,853	0,147
Endoscopy		881	708	173	26	9.698	0,965	0,804	0,877	0,981	0,019

**Table 5:** Result evaluation for Level II (shrink and classic case/SVM).

Subclass/level 2										
	Shrink case					Classic case				
	*R*	*P*	*F*	Acc	Err	*R*	*P*	*F*	Acc	Err
*Ultrasonography (document number)*										
Liver (784)	0,610	0,398	0,481	0,800	0,200	0,749	0,528	0,619	0,860	0,140
Biliary system (957)	0,416	0,360	0,386	0,753	0,247	0,572	0,436	0,495	0,783	0,217
Kidney and urinary system (536)	0,806	0,635	0,711	0,932	0,068	0,942	0,813	0,873	0,971	0,029
Gynecologic (942)	0,781	0,738	0,759	0,909	0,091	0,908	0,871	0,889	0,958	0,042
Thyroid (851)	0,805	0,752	0,778	0,924	0,076	0,825	0,794	0,809	0,936	0,064
Breast (531)	0,793	0,594	0,679	0,923	0,077	0,904	0,769	0,831	0,962	0,038
Vascular Doppler (543)	0,888	0,666	0,761	0,941	0,059	0,866	0,721	0,787	0,950	0,050

*X-ray (document number)*										
Chest (849)	0,802	0,629	0,705	0,892	0,108	0,888	0,852	0,870	0,957	0,043
Abdomen (1057)	0,729	0,634	0,678	0,861	0,139	0,855	0,762	0,806	0,917	0,083
Spine (946)	0,785	0,521	0,626	0,831	0,169	0,889	0,774	0,827	0,933	0,067
Limbs (612)	0,822	0,403	0,541	0,838	0,162	0,990	0,774	0,869	0,965	0,035
Esophagus and stomach (1224)	0,714	0,679	0,696	0,855	0,145	0,905	0,881	0,893	0,949	0,051
Large and small bowels (574)	0,796	0,603	0,686	0,921	0,079	0,911	0,944	0,927	0,984	0,016

## References

[1] Baruch Jordon J. (1965). Hospital automation via computer time-sharing. *Computers in Biomedical Research*.

[2] Morris F., Collen B., Marion J. (2015). *The History of Medical Informatics in the United States*.

[3] Yang Y. (1999). An evaluation of statistical approaches to text categorization. *Information Retrieval Journal*.

[4] Farkas R., Szarvas G. (2008). Automatic construction of rule-based ICD-9-CM coding systems. *BMC Bioinformatics*.

[5] Gao H., Aiello Bowles E. J., Carrell D., Buist D. S. M. (2015). Using natural language processing to extract mammographic findings. *Journal of Biomedical Informatics*.

[6] Sarker A., Gonzalez G. (2015). Portable automatic text classification for adverse drug reaction detection via multi-corpus training. *Journal of Biomedical Informatics*.

[7] Kotfila C., Uzuner O. (2015). A systematic comparison of feature space effects on disease classifier performance for phenotype identification of five diseases. *Journal of Biomedical Informatics*.

[8] Demner-Fushman D., Chapman W. W., McDonald C. J. (2009). What can natural language processing do for clinical decision support?. *Journal of Biomedical Informatics*.

[9] Nadkarni P. M., Ohno-Machado L., Chapman W. W. (2011). Natural language processing: an introduction. *The Journal of the American Medical Informatics Association*.

[10] Friedman C., Elhadad N. (2013). Natural language processing in health care and biomedicine. *Biomedical Informatics*.

[11] Saeys Y., Inza I., Larrañaga P. (2007). A review of feature selection techniques in bioinformatics. *Bioinformatics*.

[12] Sonel A. F., Good C. B., Rao H. (2006). *Use of REMIND Artificial Intelligence Software for Rapid Assessment of Adherence to Disease Specific Management Guidelines in Acute Coronary Syndromes*.

[13] Pollettini J. T., Panico S. R. G., Daneluzzi J. C., Tinós R., Baranauskas J. A., Macedo A. A. (2012). Using machine learning classifiers to assist healthcare-related decisions: classification of electronic patient records. *Journal of Medical Systems*.

[14] Korde V. (2012). Text classification and classifiers. *International Journal of Artificial Intelligence & Applications (IJAIA)*.

[15] Revathy N., Amalraj D. (2011). Accurate cancer classification using expressions of very few genes. *International Journal of Computer Applications*.

[16] Marafino B. J., Davies J. M., Bardach N. S., Dean M. L., Dudley R. A. (2014). N-gram support vector machines for scalable procedure and diagnosis classification, with applications to clinical free text data from the intensive care unit. *Journal of the American Medical Informatics Association*.

[17] Guyon I., Weston J., Barnhill S., Vapnik V. (2002). Gene selection for cancer classification using support vector machines. *Machine Learning*.

[18] Aronson H. (1990). *Georgian: A Reading Grammar*.

[19] Tanushi H., Dalianis H., Duneld H., Kvist M., Skeppstedt M., Velupillai S. Negation scope delimitation in clinical text using three approaches: NegEx, PyConTextNLP and SynNeg.

[20] Luo G., Liu J., Yang C. C., Deléger L., Grouin C. Detecting negation of medical problem in French clinical notes.

[21] Sebastiani F. (2002). Machine learning in automated text categorization. *ACM Computing Surveys*.

[22] Lindberg D. A. B., Humphreys B. L., McCray A. T. (1993). The unified medical language system. *Methods of Information in Medicine*.

[23] Aronson AR. Effective mapping of biomedical text to the UMLS Metathesaurus: the MetaMap program.

[24] Manning C., Raghavan P., Shutze H. (2008). *Introduction to Iinformatiion Retrieval*.

[25] Hull D. A., Grefenstette G. (1996). *A Detailed Analysis of English Stemming Algorithms*.

[26] Porter M. F. Stemming algorithms for various European languages. http://snowball.tartarus.org/texts/stemmersoverview.html.

[27] Khachidze M., Vardanidze M., Dzamashvili G. Georgian language based document classification method development.

[28] Khachidze M., Tsintsadze M., Archuadze M., Besiashvili G. (2015). Concept pattern based text classification system development for Georgian text based. *Baltic Journal of Modern Computing*.

[29] Salton G., Buckley C. (1988). Term-weighting approaches in automatic text retrieval. *Information Processing and Management*.

[30] Heaps H. S. (1978). *Information Retrieval: Computational and Theoretical Aspects*.

[31] Yang Y., Pedersen J. O. A comparative study on feature selection in text categorization.

[32] Ng A., Jordan A. (2002). On discriminative vs. generative classifiers: a comparison of logistic regression and naive bayes. *Advances in Neural Information Processing Systems*.

[33] Cristianini N., Shawe-Taylor J. (2000). *An Introduction to Support Vector Machines and other Kernel-Based Learning Methods*.

